# Supplementary material for: From sequence to enzyme mechanism using multi-label machine learning
Source: BMC Bioinformatics. 2014 May 19;15:150. doi: 10.1186/1471-2105-15-150 (PMC4229970; doi:10.1186/1471-2105-15-150)
Supplement: Additional file 2 — Java code of ml2db. Additional file ml2db_code.tar.gz contains the Java source code to run the multi-label machine learning experiments and save the results to database. The code’s Javadoc is included. [file 1471-2105-15-150-S2.zip › additional file 2/ml2db/ml2db/doc/main/ml2db/weka/Arff.html]

Arff


JavaScript is disabled on your browser.


- Overview
- Package
- Class
- Use
- Tree
- Deprecated
- Index
- Help

- Prev Class
- Next Class

- Frames
- No Frames

- All Classes

- Summary:
- Nested |
- Field |
- Constr |
- Method

- Detail:
- Field |
- Constr |
- Method


main.ml2db.weka

## Class Arff

- java.lang.Object
- - uk.ac.standrews.utils.main.Initialised
  - - main.ml2db.weka.Arff

- All Implemented Interfaces:
  :   uk.ac.standrews.utils.main.Initialisable

  Direct Known Subclasses:
  :   MulanArff

  ---

    

  ```
  public class Arff
  extends uk.ac.standrews.utils.main.Initialised
  ```

  Creates an ARFF file: checks file properties, loads data from database, fills
  and writes to file the ARFF attributes, classes and instances, generates
  auxiliary files if needed.

  Version:
  :   22 Apr 2010

  Author:
  :   Luna De Ferrari ldf(at)st-andrews.ac.uk

- - ### Field Summary

    - ### Fields inherited from class uk.ac.standrews.utils.main.Initialised

      `m_initialisationIsCorrect`
  - ### Constructor Summary

    Constructors

    Constructor and Description

    `Arff(java.lang.String dataPropsFilePath,
    DbManagers dbMan)`

    `Arff(java.lang.String dataPropsFilePath,
    DbManagers dbMan,
    boolean massive)`
  - ### Method Summary

    Methods

    | Modifier and Type | Method and Description |
    | --- | --- |
    | `void` | `checkAndInitialise(java.lang.String dataPropsFilePath, DbManagers dbMan, boolean massive)` |
    | `boolean` | `fillArff()` Loads instances, but does not write the arff file or arff database record. |
    | `int` | `generateAndWriteArffFile()` The core of the method to write Weka style ARFF files: save properties to experiment table, load data from data database and save arff to file |
    | `java.lang.String` | `getArffPath()` |
    | `int` | `getArffRecordId()` |
    | `ArffPropsFromFile` | `getDataArffProperties()` |
    | `uk.ac.standrews.utils.main.database.DbManager` | `getDataDbManager()` |
    | `java.lang.String` | `getDataPropsFilePath()` |
    | `DataSetManager` | `getDataSetManager()` |
    | `uk.ac.standrews.utils.main.database.DbConn` | `getDbConn()` |
    | `DbManagers` | `getDbManagers()` |
    | `static java.lang.String` | `getHeaderString(java.lang.String datasetToString)` Returns the header part of the arff representation (everything before the \@data label) |
    | `uk.ac.standrews.utils.main.database.DbReader` | `getInputDbReader()` |
    | `weka.core.Instances` | `getInstances()` |
    | `static java.lang.String` | `getInstancesString(java.lang.String datasetToString)` Returns the instances part of the arff representation (everything after the \@data label) |
    | `uk.ac.standrews.utils.main.database.DbManager` | `getResultsDbManager()` |
    | `static void` | `main(java.lang.String[] args)` |
    | `static int` | `run(java.lang.String arffPropsPath, DbManagers dbMan, boolean massive)` |
    | `int` | `setArffRecordId(int arffRecordId)` Sets the arff record id and returns it for further use. |
    | `void` | `setDataSetManager()` |
    | `boolean` | `setup()` Sets up the data sources, destination etc. |
    | `int` | `writeArff(boolean set, boolean filled)` |

    - ### Methods inherited from class uk.ac.standrews.utils.main.Initialised

      `initialisationIsCorrect, setInitialisation, setInitialisationIsCorrect, setInitialisationNotCorrect`
    - ### Methods inherited from class java.lang.Object

      `equals, getClass, hashCode, notify, notifyAll, toString, wait, wait, wait`

- - ### Constructor Detail


    - #### Arff

      ```
      public Arff(java.lang.String dataPropsFilePath,
          DbManagers dbMan)
      ```


    - #### Arff

      ```
      public Arff(java.lang.String dataPropsFilePath,
          DbManagers dbMan,
          boolean massive)
      ```
  - ### Method Detail


    - #### checkAndInitialise

      ```
      public void checkAndInitialise(java.lang.String dataPropsFilePath,
                            DbManagers dbMan,
                            boolean massive)
      ```


    - #### fillArff

      ```
      public boolean fillArff()
      ```

      Loads instances, but does not write the arff file or arff database
      record.


    - #### generateAndWriteArffFile

      ```
      public int generateAndWriteArffFile()
      ```

      The core of the method to write Weka style ARFF files: save properties to
      experiment table, load data from data database and save arff to file

      Returns:
      :   the arff file record id in the database, or -1 if the file could
          not be written.


    - #### getArffPath

      ```
      public java.lang.String getArffPath()
      ```


    - #### getArffRecordId

      ```
      public int getArffRecordId()
      ```


    - #### getDataArffProperties

      ```
      public ArffPropsFromFile getDataArffProperties()
      ```


    - #### getDataDbManager

      ```
      public uk.ac.standrews.utils.main.database.DbManager getDataDbManager()
      ```


    - #### getDataPropsFilePath

      ```
      public java.lang.String getDataPropsFilePath()
      ```


    - #### getDataSetManager

      ```
      public DataSetManager getDataSetManager()
      ```


    - #### getDbConn

      ```
      public uk.ac.standrews.utils.main.database.DbConn getDbConn()
      ```


    - #### getDbManagers

      ```
      public DbManagers getDbManagers()
      ```


    - #### getInputDbReader

      ```
      public uk.ac.standrews.utils.main.database.DbReader getInputDbReader()
      ```


    - #### getInstances

      ```
      public weka.core.Instances getInstances()
      ```


    - #### getResultsDbManager

      ```
      public uk.ac.standrews.utils.main.database.DbManager getResultsDbManager()
      ```


    - #### setArffRecordId

      ```
      public int setArffRecordId(int arffRecordId)
      ```

      Sets the arff record id and returns it for further use.

      Parameters:
      :   `arffRecordId` - the table id for this arff file details

      Returns:
      :   the table id for this arff file details


    - #### setDataSetManager

      ```
      public void setDataSetManager()
      ```


    - #### setup

      ```
      public boolean setup()
      ```

      Sets up the data sources, destination etc. and loads the data, but does
      not fill the dataset yet, nor write it.


    - #### writeArff

      ```
      public int writeArff(boolean set,
                  boolean filled)
      ```


    - #### getHeaderString

      ```
      public static java.lang.String getHeaderString(java.lang.String datasetToString)
      ```

      Returns the header part of the arff representation (everything before the
      \@data label)

      Parameters:
      :   `datasetToString` - the result of an Instances.toString() or the content of an
          arff file.

      Returns:
      :   the the header part of the arff representation (everything before
          the \@data label)


    - #### getInstancesString

      ```
      public static java.lang.String getInstancesString(java.lang.String datasetToString)
      ```

      Returns the instances part of the arff representation (everything after
      the \@data label)

      Parameters:
      :   `datasetToString` - the result of an Instances.toString() or the content of an
          arff file.

      Returns:
      :   the instances part of the arff representation (everything after
          the \@data label)


    - #### main

      ```
      public static void main(java.lang.String[] args)
      ```


    - #### run

      ```
      public static int run(java.lang.String arffPropsPath,
            DbManagers dbMan,
            boolean massive)
      ```


- Overview
- Package
- Class
- Use
- Tree
- Deprecated
- Index
- Help

- Prev Class
- Next Class

- Frames
- No Frames

- All Classes

- Summary:
- Nested |
- Field |
- Constr |
- Method

- Detail:
- Field |
- Constr |
- Method
